# Safety of Ticagrelor Compared to Clopidogrel after Prehospital Initiation of Treatment

**DOI:** 10.1055/s-0038-1673389

**Published:** 2018-10-11

**Authors:** Thomas O. Bergmeijer, Mathijs van Oevelen, Paul W. A. Janssen, Thea C. Godschalk, Robert A. Lichtveld, Johannes C. Kelder, Michiel Voskuil, Arend Mosterd, Gilles Montalescot, Jurriën M. ten Berg

**Affiliations:** 1 Department of Cardiology, St. Antonius Hospital, Nieuwegein, The Netherlands; 2 Regional Ambulance Service Utrecht (RAVU), Bilthoven, The Netherlands; 3 Division of Heart and Lungs, Department of Cardiology, UMC Utrecht, The Netherlands; 4 Department of Cardiology, Meander Medical Center, Amersfoort, The Netherlands; 5 ACTION Study Group, UPMC Sorbonne Universités, Pitié-Salpêtrière Hospital (AP-HP), Paris, France

**Keywords:** myocardial infarction, ticagrelor, clopidogrel, prehospital emergency care, hemorrhage

## Abstract

**Objectives** The objective of this registry was to study the safety of prehospital initiation of ticagrelor compared with clopidogrel.

**Background** Ticagrelor has replaced clopidogrel in many hospitals as the routinely used antiplatelet drug in patients with ST-segment elevation myocardial infarction (STEMI). Nevertheless, in the PLATelet inhibition and patient Outcomes (PLATO) trial, ticagrelor was associated with an increase in non-CABG (non–coronary artery bypass grafting)-related major bleeding. Data comparing the safety of ticagrelor and clopidogrel after prehospital initiation of treatment are not available.

**Methods** A retrospective, multicenter registry was performed. Selection criteria were the administration of a prehospital loading dose of ticagrelor or clopidogrel according to the ambulance STEMI treatment protocol and the presentation to a percutaneous coronary intervention–capable hospital in our region between January 2011 and December 2012. Follow-up was performed using the electronic patient files for the time period between the antiplatelet loading dose and hospital discharge. The data were analyzed using a primary bleeding end point (any bleeding) and a secondary thrombotic end point (all-cause mortality, spontaneous myocardial infarction, definite stent thrombosis, stroke, or transient ischemic attack).

**Results** Data of 304 clopidogrel-treated and 309 ticagrelor-treated patients were available for analysis. No significant difference in bleeding rate was observed between both groups, using univariate (17.8 vs. 20.1%; *p* = 0.47; odds ratio, 1.16 [95% confidence interval, 0.78–1.74]) and multivariate ( *p* = 0.42) analysis. Also for the secondary thrombotic end point (6.3 vs. 4.9%, *p* = 0.45), no significant differences were observed.

**Conclusion** In this real-world registry, no significant differences in bleeding or thrombotic event rate were found between ticagrelor and clopidogrel after prehospital initiation of treatment.

## Introduction

Dual antiplatelet therapy (DAPT) plays a major role in the acute treatment of ST-segment elevation myocardial infarction (STEMI), as the process of thrombus formation during STEMI is strongly platelet driven. 1 2 3 DAPT reduces the number of atherothrombotic events in patients with acute myocardial infarction and in patients undergoing percutaneous coronary intervention (PCI). 3 4 5 6 The early use of DAPT in STEMI patients also prevents the occurrence of stent thrombosis, which is particularly relevant because stent thrombosis is associated with a high mortality rate. 7 8 9 The current STEMI guidelines recommend the early administration of DAPT, as soon as the STEMI diagnosis is established. 10 11

Until a few years ago, the cyclooxygenase (COX)-1 inhibitor aspirin and the adenosine diphosphate (ADP) receptor antagonist clopidogrel were the treatment of choice. With the introduction of prasugrel and ticagrelor, two more potent oral antiplatelet drugs became available. Tested in large cohorts of patients with acute coronary syndrome, both drugs proved to be more effective in the prevention of recurrent atherothrombotic events. 12 13 Also, both drugs showed a faster onset of action compared with clopidogrel. 14 15 16 17 Keeping in mind that acute myocardial infarction results in an increased platelet aggregation and that the risk of stent thrombosis is the highest in the first hours after primary PCI, these higher efficacy and faster onset of action make prasugrel and ticagrelor preferred drugs nowadays over clopidogrel in the treatment of STEMI patients. 10 14 18 19 In the Netherlands, prasugrel and ticagrelor have replaced clopidogrel in the prehospital STEMI treatment protocols, following the publication of the 2012 European Society of Cardiology (ESC) STEMI guideline. 20 The downside of these drugs is that the bleeding risk is higher, which might be even more evident when used in specific subgroups of patients (i.e., in the elderly), related to the risk of periprocedural bleeding, or in combination with other anticoagulants, such as heparin and glycoprotein IIb/IIIa inhibitors (GPI). The available data describing the bleeding risk are limited, because most anticoagulants were only tested in combination with clopidogrel. 12 13 21 22 Moreover, although the STEMI diagnosis made in the ambulance is correct in the large majority of patients, some patients will be misdiagnosed and treated with antiplatelet treatment in the absence of a myocardial infarction.

The aim of this registry was to study the safety of prehospital administration of ticagrelor in comparison to clopidogrel in a real-world setting.

## Methods

This study was designed as a retrospective, observational, multicenter registry. Patients were selected from the digital records of the regional ambulance service Utrecht (RAVU), an organization providing ambulance service to 1.3 million residents in the province of Utrecht in the Netherlands. All consecutive patients between January 1, 2011, and December 31, 2012, who received a loading dose of either clopidogrel or ticagrelor and were subsequently transported to one of the three PCI-capable hospitals in the area were selected.

The RAVU changed their STEMI treatment protocol on December 15, 2011: before this date, clopidogrel was administered in all patients suspected of having a STEMI, while from December 15, 2011, on, ticagrelor was administered in these patients. There were no differences between both protocols regarding which patients were eligible for treatment. Both the RAVU's electronic patient file and the hospital's electronic patient file were used to collect baseline characteristics, diagnosis, treatment, and outcome data. Patients without in-hospital follow-up data were excluded from the analysis. The local ethics committee provided a waiver for obtaining written informed consent, based on the noninvasive character of the study.

### End Points

The primary end point was the occurrence of any bleeding event between the administration of the clopidogrel or ticagrelor loading dose in the ambulance and hospital discharge. Bleeding events were classified using the Bleeding Academic Research Consortium (BARC) bleeding criteria. 23 As a secondary end point, bleeding was evaluated for different subgroups of bleeding, i.e., combined BARC 2–5 bleeding, periprocedural bleeding related to angiography or PCI, bleeding requiring invasive intervention (surgery or endoscopy with intervention), and bleeding requiring transfusion.

Additionally, a thrombotic end point was analyzed, consisting of all-cause mortality, nonfatal myocardial infarction, definite stent thrombosis, and nonfatal stroke or transient ischemic attack (TIA), both as separate events and as a composite end point. Myocardial infarction was defined as type 1 spontaneous myocardial infarction according to the 2012 universal definition of myocardial infarction. 24 Definite stent thrombosis was classified as angiographically confirmed stent thrombosis, according to the Academic Research Consortium criteria. 25 Stroke and TIA were defined as any new neurological deficit, lasting for more than 24 hours (stroke) or less than 24 hours (TIA) and described as such by a neurologist.

Outcome events were adjudicated by the first two authors (T. B. and M. v. O.), and the last author (J. M. t. B.) if there was no agreement.

### Sample Size and Statistical Analysis

The sample size of our registry was based on a timeframe, chosen to obtain two comparably sized groups, rather than a calculated sample size. Reason was that there were no detailed data available describing in-hospital bleeding rates in an all-comer population after prehospital administration of a clopidogrel or ticagrelor loading dose. We estimated that this would yield a total number of patients that would be substantial enough to draw relevant conclusions, but would also be feasible for the follow-up data collection. Therefore, this analysis should be considered as exploratory.

Patient characteristics were compared using a two-tailed Student's *t* -test for continuous variables and a two-tailed Pearson's chi-square test for binary and categorical variables. Statistical analyses were performed using SPSS software (version 22, IBM, USA). A *p* -value < 0.05 was considered statistically significant. Baseline variables with unequal distribution between both groups and which were considered relevant for our analysis were used for multivariate analysis using logistic regression. A likelihood ratio test was used to calculate the interaction *p* -value.

## Results

### Study Cohorts

A total of 704 patients received a clopidogrel or ticagrelor loading dose in the ambulance before or during transport to a PCI-capable hospital. Of those patients, diagnosis and complete in-hospital follow-up was obtained in 613 patients: 304 patients received a 600-mg clopidogrel loading dose (cohort prior to December 15, 2011) and 309 patients received a 180-mg ticagrelor loading dose (cohort starting December 15, 2011). The remaining 91 patients were transferred to a hospital not participating in this registry and were therefore excluded from the analysis.

Baseline characteristics for the clopidogrel and ticagrelor treated groups are listed in Table 1 . Both treatment groups were well balanced according to age, gender, and cardiovascular risk factors. Patients were hospitalized for an average of 5.9 days. A cardiac-related condition was diagnosed in 554 patients (90.4%), and in 505 patients (82.4%) an acute myocardial infarction was diagnosed. Coronary angiography and PCI with stent implantation were performed in 540 (88.1%) and 409 (66.7%) patients, respectively. In patients with myocardial infarction, those percentages were 97 and 88.9%. The use of the radial compared with the femoral arterial access site was higher in the ticagrelor-treated patient group (41.9 vs. 28.3%, *p* < 0.001), as was the implantation of a drug-eluting stent (DES) (66.7 vs. 41.9%, *p* < 0.001). Thrombolysis in myocardial infarction (TIMI) 3 flow after intervention was comparable between both groups (88.8 vs. 90.5%, *p* = 0.56).

**Table 1:** Baseline characteristics

	Clopidogrel ( *n* = 304)	Ticagrelor ( *n* = 309)	*p* -Value
**General characteristics**			
Age (y)	63.2 ± 13.8	62.6 ± 13.5	0.58
Male gender	214/304 (70.4)	226/309 (73.1)	0.45 a
Body mass index (kg/m ^2^ )			
<20	9/304 (3.0)	9/309 (2.9)	0.40
20–25	108/304 (35.5)	94/309 (30.4)	
≥25	187/304 (61.5)	206/309 (66.7)	
Hypertension	135/304 (44.4)	124/309 (40.1)	0.28
Dyslipidemia	125/304 (41.1)	119/309 (38.5)	0.51
Current smoker	105/304 (34.5)	109/309 (35.3)	0.85
Diabetes mellitus	44/304 (14.5)	47/309 (15.2)	0.80
Family history of cardiovascular disease	107/304 (35.2)	111/309 (35.9)	0.85
Recent bleeding	4/304 (1.3)	0/309 (0)	0.06
Previous stroke or TIA	18/304 (5.9)	16/309 (5.2)	0.69
Creatinine value	89.7 ± 46.9	88.3 ± 50.4	0.73
Renal failure (eGFR < 45 mL/min)	19/279 (6.8)	19/301 (6.3)	0.81
**Antiplatelet/anticoagulant use before loading dose**			
Acetylsalicylic acid	57/304 (18.8)	69/309 (22.3)	0.27
Clopidogrel	5/304 (1.6)	5/309 (1.6)	1.00
Ticagrelor	0/304 (0)	2/309 (0.6)	0.50
Dipyridamole	7/304 (2.3)	2/309 (0.6)	0.10
Vitamin K antagonists	17/304 (5.6)	15/309 (4.9)	0.68
**Antiplatelet/anticoagulant at admission**			
Acetylsalicylic acid in ambulance	291/304 (95.7)	296/309 (95.8)	0.97
GPI use			
No GPI used	194/304 (63.8)	194/309 (62.8)	<0.001 a
Started in ambulance	0/304 (0)	52/309 (16.8)	
Started during CAG/PCI	110/304 (36.2)	63/309 (20.4)	
Bivalirudin use			
No bivalirudin used	303/304 (99.7)	243/309 (78.6)	<0.001
Started in ambulance	0/304 (0)	55/309 (17.8)	
Started during CAG/PCI	1/304 (0.3)	11/309 (3.6)	
Heparin use in ambulance	294/304 (96.7)	235/309 (76.1)	<0.001
Heparin or bivalirudin	294/304 (96.7)	290/309 (93.9)	0.10
Fentanyl in ambulance	84/304 (27.6)	95/309 (30.7)	0.40
**Hospitalization details**			
Time between FMC and hospital arrival (min)	36:08 ± 11:49	37:34 ± 13:13	0.17
Hospitalization length (d)	5.9 ± 4.8	5.9 ± 4.6	0.88
Cardiac diagnosis	274/304 (90.1)	280/309 (90.6)	0.84
Myocardial infarction	246/304 (80.9)	259/309 (83.8)	0.35
STEMI	225/304 (74.0)	246/309 (79.6)	0.10
Non-STEMI	21/304 (6.9)	13/309 (4.2)	0.14
CK max	1,574 ± 1,801	1,597 ± 1,631	0.89
Unstable angina	13/304 (4.3)	5/309 (1.6)	0.05
Other cardiac diagnosis	15/304 (4.9)	16/309 (5.2)	0.89
**Intervention**			
Coronary angiography	267/304 (87.8)	273/309 (88.3)	0.84
PCI with stent implantation	199/304 (65.5)	210/309 (68.0)	0.51
DES implanted	80/171 (41.9)	136/204 (66.7)	<0.001 a
Arterial access site			
Femoral	183/267 (68.5)	154/273 (56.4)	<0.01 a
Radial	73/267 (27.3)	112/273 (41.0)	
Other/unknown	11/267 (4.1)	7/273 (2.6)	
Sheath diameter			
6 French	195/204 (95.6)	236/242 (97.5)	0.26
7 or 8 French	9/204 (4.4)	6/242 (2.5)	
Thrombosuction	108/241 (44.8)	121/246 (49.2)	0.33
TIMI 3 flow after procedure	174/196 (88.8)	200/221 (90.5)	0.56
CABG	14/304 (4.6)	15/309 (4.9)	0.89

Abbreviations: CABG, coronary artery bypass grafting; CAG, coronary angiography; CK, creatinine kinase; DES, drug-eluting stent; eGFR, estimated glomerular filtration rate; FMC, first medical contact; GPI, glycoprotein IIb/IIIa inhibitors; PCI, percutaneous coronary intervention; STEMI, ST-segment elevation myocardial infarction; TIA, transient ischemic attack; TIMI, thrombolysis in myocardial infarction.

Note: All results are expressed as mean ± SD or number/total number (%).

a Included in logistic regression model.

In the ticagrelor-treated patient group, 90 patients (29.1%) were treated according to the EUROMAX study protocol. In this randomized trial, the prehospital administration of heparin plus optional GPI (tirofiban) was compared with the use of bivalirudin in STEMI patients. 26 Nevertheless, the use of heparin or bivalirudin was not statistically different between both our study groups (96.7 vs. 93.9%, *p* = 0.10), although the prehospital administration of heparin was more frequent in the clopidogrel-treated patients compared with the ticagrelor-treated group (96.7 vs. 76.1%, respectively, *p* < 0.01), while 17.8% of ticagrelor-treated patients received prehospital bivalirudin infusion. The total number of patients who were treated with a GPI was not significantly different between both groups, although prehospital administration was performed in the ticagrelor-treated group only.

### Outcome Events

**Fig. 1:**
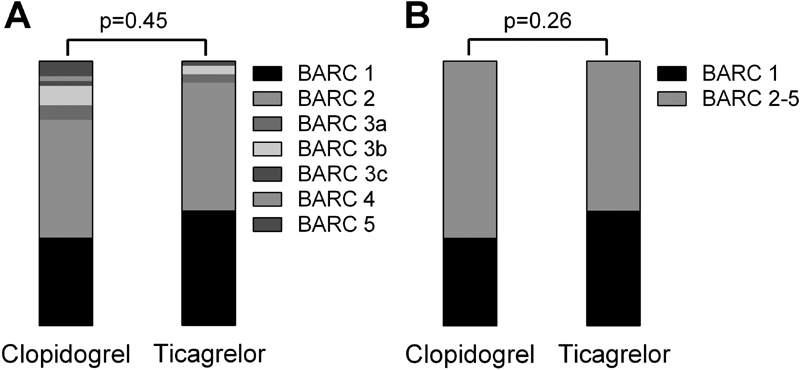
Distribution of BARC bleeding scores. Distribution of BARC bleeding scores for all BARC subgroups ( **A** ) and for combined BARC 2–5 bleeding ( **B** ). BARC, Bleeding Academic Research Consortium.

Between administration of the antiplatelet loading dose and hospital discharge, 17.8% of clopidogrel-treated patients suffered a bleeding event, as compared with 20.1% of the ticagrelor-treated patients ( *p* = 0.47, Table 2 ). After multivariate analysis, adjusting for baseline differences between the clopidogrel and ticagrelor group—gender, GPI use, arterial access site, and the use of one or more DESs—“any bleeding” was still not significantly different between both groups ( *p* = 0.42). The number of bleeding events requiring intervention was higher in the clopidogrel-treated patients (3.3 vs. 0.6%, *p* = 0.02), which could not be attributed to differences in arterial access site or GPI use. No significant differences were found when bleeding was classified according to BARC bleeding types or for combined BARC 2–5 bleeding ( Table 2 and Fig. 1 ). Multivariate analysis using baseline characteristics predictive and relevant for bleeding events ( Appendix Table 1 )—age, gender, arterial access site, and estimated glomerular filtration rate < 45mL/min—showed no significant difference between the clopidogrel- and ticagrelor-treated group ( *p* = 0.36). Bivalirudin use could not be accounted for in the multivariate analysis, because it was only administered in the ticagrelor-treated group in patients in the EUROMAX trial. Therefore, we performed the same univariate and multivariate analysis in a selection of patients treated with heparin in the ambulance, to compensate for the effect of the EUROMAX trial, which did not result in any statistically significant differences between both groups (data not shown). This was the same when all patients participating in the EUROMAX study were omitted from the analysis.

**Table 2:** Bleeding events during hospitalization according to treatment group

	Clopidogrel ( *n* = 304)	Ticagrelor ( *n* = 309)	OR (95% CI)	*p* -Value	Adjusted OR (95% CI) a	*p* -Value a
All bleeding events	54 (17.8)	62 (20.1)	1.16 (0.78–1.74)	0.47	1.20 (0.77–1.88)	0.42
BARC 1	18 (5.9)	27 (8.7)	1.52 (0.82–2.82)	0.18	1.54 (0.79–2.99)	0.21
BARC 2–5	36 (11.8)	35 (11.3)	0.95 (0.58–1.56)	0.84	0.98 (0.57–1.69)	0.95
BARC 2	24 (7.9)	30 (9.7)	1.25 (0.72–2.20)	0.43	1.42 (0.77–2.62)	0.26
BARC 3	8 (2.6)	4 (1.3)	0.49 (0.15–1.63)	0.23	0.31 (0.06–1.47)	0.14
BARC 3a	3 (1.0)	2 (0.6)	0.65 (0.11–3.94)	0.64	1.00	1.00
BARC 3b	4 (1.3)	2 (0.6)	0.49 (0.09–2.69)	0.45	0.74 (0.13–4.16)	0.73
BARC 3c	1 (0.3)	0 (0)	–	0.50	1.00	0.99
BARC 4	1 (0.3)	0 (0)	–	0.50	1.00	0.99
BARC 5	3 (1.0)	1 (0.3)	0.33 (0.03–3.15)	0.37	0.32 (0.03–3.31)	0.34
**Subgroups of bleeding**						
Bleeding related to CAG/PCI	39 (12.8)	46 (14.9)	1.19 (0.75–1.88)	0.46	1.24 (0.74–2.07)	0.42
Requiring intervention	10 (3.3)	2 (0.6)	0.19 (0.04–0.88)	0.02	0.11 (0.01–0.86)	0.03
Requiring transfusion	7 (2.3)	3 (1.0)	0.42 (0.11–1.62)	0.22	0.19 (0.02–1.59)	0.13

Abbreviations: BARC, Bleeding Academic Research Consortium; CAG, coronary angiography; CI, confidence interval; GPI, glycoprotein IIb/IIIa inhibitor; OR, odds ratio; PCI, percutaneous coronary intervention.

a Adjusted for gender, arterial access site, GPI use, and implantation of one or more DES stents.

**Appendix Table 1:** Univariate predictors for bleeding

	Bleeding ( *n* = 116)	No bleeding ( *n* = 497)	*p* -Value
**General characteristics**			
Age (y)	67.5 ± 12.7	62.8 ± 13.6	<0.001 a
Male gender	70/116 (60.3)	370/497 (74.4)	<0.01 a
Body mass index (kg/m ^2^ )			
<20	5/116 (5.2)	12/497 (2.4)	0.05
20–25	46/116 (39.7)	156/497 (31.4)	
≥25	64/116 (55.2)	329/497 (66.2)	
Hypertension	58/116 (50.0)	201/497 (40.4)	0.06
Dyslipidemia	39/116 (33.6)	205/497 (41.2)	0.13
Current smoker	30/116 (25.9)	184/497 (37.0)	0.02
Diabetes mellitus	19/116 (16.4)	72/497 (14.5)	0.61
Family history of cardiovascular disease	43/116 (37.1)	175/497 (35.2)	0.71
Recent bleeding	2/116 (1.7)	2/497 (0.4)	0.16
Previous stroke or TIA	8/116 (6.9)	26/497 (5.2)	0.48
Creatinine value	102.6 ± 96.5	85.7 ± 25.5	0.001 a
Renal failure (eGFR < 45)	14/113 (12.4)	24/467 (5.1)	0.005
**Antiplatelet/anticoagulant use before loading dose**			
Acetylsalicylic acid	35/116 (30.2)	91/497 (18.2)	<0.01
Clopidogrel	6/116 (5.2)	4/497 (0.8)	<0.01
Ticagrelor	0/116 (0)	2/497 (0.4)	0.66
Dipyridamole	3/116 (2.6)	6/497 (1.2)	0.23
Vitamin K antagonists	8/116 (6.9)	24/497 (4.8)	0.37
**Antiplatelet/anticoagulant at admission**			
Acetylsalicylic acid in ambulance	109/116 (94.0)	478/497 (96.2)	0.20
GPI use			
No use	70/116 (60.3)	318/497 (64.0)	0.48
In ambulance	13/116 (11.2)	39/497 (8.7)	
During CAG/PCI	33/116 (28.4)	140/497 (28.2)	
Bivalirudin use			
No use	107/116 (92.2)	439/497 (88.3)	0.42
In ambulance	8/116 (6.9)	47/497 (9.5)	
During CAG/PCI	1/116 (0.9)	11/497 (2.2)	
Heparin use in ambulance	107/116 (92.2)	422/497 (84.9)	0.04
Heparin or bivalirudin	115/116 (99.1)	469/497 (94.4)	0.03
Fentanyl use in ambulance	38/116 (32.8)	141/497 (28.4)	0.35
**Hospitalization details**			
Time between FMC and hospital arrival (min)	37:44 ± 13:27	36:40 ± 12:21	0.42
Hospitalization length (d)	6.8 ± 4.7	5.7 ± 4.7	0.02
Cardiac diagnosis	112/116 (96.6)	442/497 (88.9)	0.01
Myocardial infarction	104/116 (89.7)	401/497 (80.7)	0.02
STEMI	97/116 (83.6)	374/497 (75.3)	0.05
Non-STEMI	7/116 (6.0)	27/497 (5.4)	0.80
CK max	1,270 ± 1,461	1,671 ± 1,764	0.04
Unstable angina	4/116 (3.4)	14/497 (2.8)	0.45
Other cardiac diagnosis	4/116 (3.4)	27/497 (5.4)	0.38
**Intervention**			
Coronary angiography	65/116 (56.0)	432/497 (86.9)	0.06
PCI with stent implantation	78/116 (67.2)	331/497 (66.6)	0.90
DES implanted	48/77 (62.3)	168/318 (52.8)	0.13
Arterial access site			
Femoral	77/108 (71.3)	260/432 (60.2)	0.04 a
Radial	29/108 (26.9)	156/432 (36.1)	
Other/unknown	2/108 (1.9)	16/432 (3.7)	
Sheath diameter			
6 French	86/99 (86.9)	345/420 (82.1)	0.07
7 or 8 French	5/99 (5.1)	10/420 (2.4)	
Thrombosuction	34/97 (35.1)	195/390 (50.0)	0.008
TIMI 3 flow after procedure	76/84 (90.5)	298/333 (89.5)	0.79
CABG	9/116 (7.8)	20/497 (4.0)	0.09

Abbreviations: CABG, coronary artery bypass grafting; CAG, coronary angiography; CK, creatinine kinase; DES, drug-eluting stent; eGFR, estimated glomerular filtration rate; FMC, first medical contact; GPI, glycoprotein IIb/IIIa inhibitors; PCI, percutaneous coronary intervention; STEMI, ST-segment elevation myocardial infarction; TIA, transient ischemic attack; TIMI, thrombolysis in myocardial infarction.

Note: All results are expressed as mean ± SD or number/total number (%).

a Included in logistic regression model.

The composite thrombotic end point was not statistically significantly different between clopidogrel- and ticagrelor-treated patients, both in univariate analysis (6.3 vs. 4.9%, *p* = 0.45) and multivariate analysis ( *p* = 0.30) ( Table 3 ). All-cause mortality was comparable among both groups (3.3 vs. 3.2%, *p* = 0.97), while definite stent thrombosis and stroke or TIA were numerically lower in the ticagrelor-treated patient group. Myocardial infarction (not related to stent thrombosis) was not observed during in-hospital follow-up.

**Table 3:** Atherothrombotic events during hospitalization according to treatment group

	Clopidogrel ( *n* = 304)	Ticagrelor ( *n* = 309)	OR (95% CI)	*p* -Value	Adjusted OR (95% CI) a	*p* -Value a
Combined thrombotic end point	19/304 (6.3)	15/309 (4.9)	0.77 (0.38–1.54)	0.45	0.67 (0.31–1.45)	0.30
All-cause mortality	10/304 (3.3)	10/309 (3.2)	0.98 (0.40–2.40)	0.97	0.83 (0.31–2.27)	0.72
Definite stent thrombosis	7/304 (2.3)	5/309 (1.6)	0.70 (0.22–2.22)	0.54	0.78 (0.22–2.81)	0.71
Stroke or TIA	2/304 (0.7)	0/309 (0)	–	0.25	1.00	0.99
Spontaneous myocardial infarction	0/304 (0)	0/309 (0)	–	–	–	–

Abbreviations: CI, confidence interval; GPI, glycoprotein IIb/IIIa inhibitor; OR, odds ratio; TIA, transient ischemic attack.

a Adjusted for gender, arterial access site, GPI use, and implantation of one or more DES-stents.

In the PRIVATE-ATLANTIC study, the administration of morphine was related to delayed onset of antiplatelet effect of ticagrelor. 27 In our registry, 179 patients (29.2%) received fentanyl intravenously in the ambulance, which was not significantly associated with the bleeding end point, the thrombotic end point, or definite stent thrombosis, both in the clopidogrel and ticagrelor subgroups, and for the combined study cohort.

### Interaction Subgroup Analysis

**Fig. 2:**
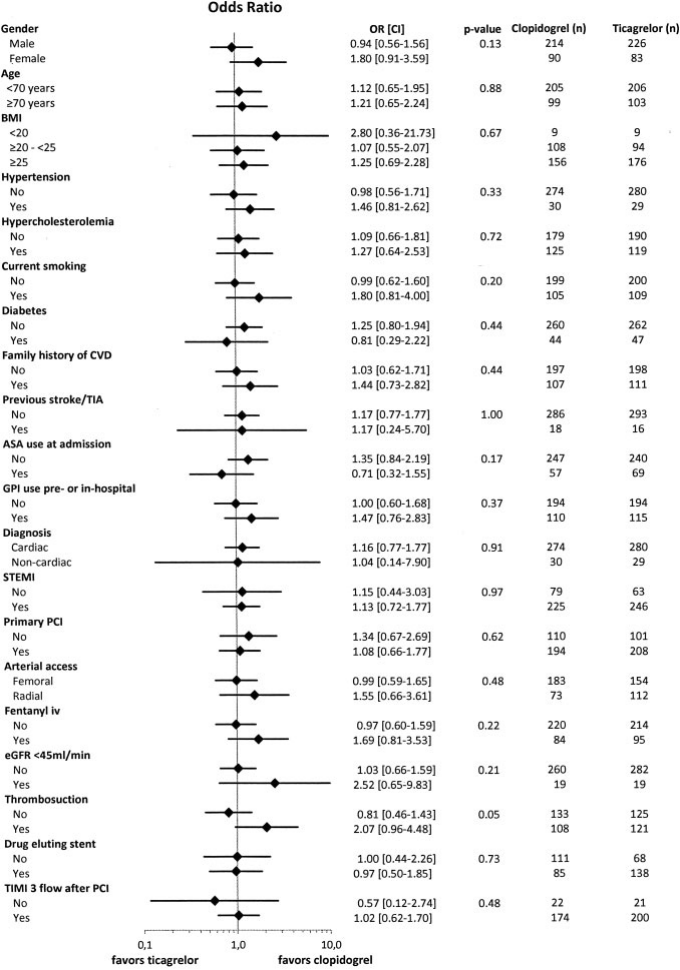
Subgroup analysis for interaction. *p* -Value is the value for interaction. ASA, acetylsalicylic acid; BMI, body mass index; CI, confidence interval; CVD, cardiovascular disease; DES, drug-eluting stent; eGFR, estimated glomerular filtration rate; GPI, glycoprotein GPIIb/IIIa inhibitor; *n* , number; OR, odds ratio; PCI, percutaneous coronary intervention; STEMI, ST-segment elevation myocardial infarction; TIA, transient ischemic attack; TIMI, thrombolysis in myocardial infarction.

In a subgroup analysis evaluating all relevant baseline and treatment variables available, no specific subgroup of patients could be identified to be at a significantly increased bleeding risk after clopidogrel or ticagrelor loading dose ( Fig. 2 ).

## Discussion

In this registry, we compared the in-hospital outcome of clopidogrel versus ticagrelor after initiation of treatment in the ambulance. Although the number of BARC 1 bleeding events was numerically higher, and thrombotic events, i.e., stent thrombosis, were lower when ticagrelor was used instead of clopidogrel, no significant differences were found.

### Advantages of Prehospital Antiplatelet Loading Dose

In observational studies, the administration of clopidogrel before the start of the primary PCI has shown to be beneficial. 3 28 29 In the randomized CIPAMI trial, which compared prehospital administration of a clopidogrel loading dose to a loading dose after diagnostic angiography in 337 STEMI patients, a nonsignificant reduction in the combined end point of death, reinfarction, and urgent target vessel revascularization was found (3.0 vs. 7.0%, *p* = 0.09), without an increase in the number of bleeding events associated with prehospital clopidogrel administration. 30 The efficacy and safety of early P2Y _12_ inhibition is confirmed by a recent meta-analysis containing 9,648 STEMI patients, performed by Bellemain-Appaix et al, showing improvement of coronary reperfusion before PCI and a reduction in major adverse cardiovascular events (MACE) and stent thrombosis (nonsignificant), without increase in major bleeding. 31

The prospective multicenter MULTIPRAC registry compared the prehospital use of clopidogrel and prasugrel in 2,053 STEMI patients. 32 Although no difference was found for clinical outcome, the percentage of patients with post-PCI ST-segment resolution of at least 50% on ECG was significantly higher in the prasugrel-treated group (65.0 vs. 71.6%, *p* = 0.005), without a significant increase in bleeding rate. An observational study performed by De Backer et al in 3,497 STEMI patients, however, found no benefit of prasugrel or ticagrelor compared with clopidogrel on early coronary reperfusion (TIMI 3 flow in the infarct-related artery) or 30 days MACE, although this strategy seemed to be safe without increase in major bleeding. 33 Our analysis has a comparable design to the study performed by De Backer et al. Most important differences are the selection of patients (all patients loaded with a P2Y _12_ inhibitor in the ambulance in our study vs. STEMI patients with less than 6 hours of symptoms and undergoing primary PCI in De Backer et al), the time since the administration of the antiplatelet loading dose (36 minutes to hospital arrival vs. ∼65–75 minutes to angiography), and sample size (304 clopidogrel-treated and 309 ticagrelor-treated patients vs. 1,532 clopidogrel-treated and 491 ticagrelor-treated patients). Nevertheless, both studies do not find a significant advantage or disadvantage in short-term clinical outcome for prehospital ticagrelor over clopidogrel.

The ATLANTIC trial, in which 1,862 STEMI patients were randomized between a prehospital and in-hospital ticagrelor loading dose, did not find improvement in reperfusion of the culprit artery associated with prehospital loading, although prehospital ticagrelor administration was associated with a lower number of definite stent thrombosis events within 24 hours after PCI (0 vs. 0.8%, *p* = 0.008). 7 A prehospital ticagrelor loading dose was not associated with an increase in major bleeding events compared with in-hospital administration. In the ATLANTIC trial, however, the time difference between prehospital and in-hospital ticagrelor administration was only 31 minutes on average.

A registry study can provide important information about real-world patient care, which a randomized controlled trial might not be able to detect. Although the study design (registry vs. randomized controlled trial) was different, our registry has similarities with the ATLANTIC trial. Most baseline characteristics are comparable and the short prehospital treatment time observed in the ATLANTIC trial was comparable with the treatment time observed in our registry (31 minutes between prehospital and in-hospital ticagrelor administration in ATLANTIC vs. 36 minutes between first medical contact and hospital arrival in our registry). The findings of our observational study strengthen the main conclusion of the ATLANTIC trial that ticagrelor is safe to use in the prehospital setting. Furthermore, the significant lower incidence of stent thrombosis related to prehospital administration of ticagrelor found in the ATLANTIC trial matches the numerical trend in our registry, although our finding was not statistically significant. Factors hampering the power of our findings are the comparison with clopidogrel (instead of comparison to placebo) and the lower number of patients. There was no signal found for a difference in mortality rate associated with early ticagrelor use in both the ATLANTIC trial and in our registry, although a recent published subgroup analysis of the ATLANTIC with French patients only—characterized by a higher rate of radial access, prehospital GPI use, and intravenous enoxaparin—showed a significant reduction in mortality associated with prehospital ticagrelor administration (1.4 vs. 3.3%, *p* = 0.01). 34 While morphine use appeared to be of importance in the ATLANTIC trial, this effect was not found for the prehospital use of fentanyl in our registry. Like with morphine, fentanyl use is associated with a delayed onset of action of oral antiplatelet agents, most likely associated with delayed gastric emptying. 35 36 37

### Disadvantages of Prehospital Antiplatelet Loading Dose

Although there is evidence to support routine prehospital administration of DAPT in STEMI patients, there are also disadvantages. It might be difficult to identify the patients with a contraindication for antiplatelet therapy in the prehospital setting, although the number of patients with an absolute contraindication is relatively low and depends on which ADP receptor antagonist is used. 38 Despite the fact that all patients in our registry received a clopidogrel or ticagrelor loading dose, according to the ambulance STEMI protocol, 23.2% of patients were eventually not diagnosed with STEMI and in a substantial 9.6% of patients no cardiac-related problem was found. Those patients experience a bleeding risk without the treatment benefit. Finally, the distance to the nearest PCI-capable hospital is generally short in the Netherlands and STEMI patients will be directly presented to the catheterization laboratory whenever possible. With a time between first medical contact and hospital arrival of just over 30 minutes, there is only limited time to win with prehospital initiation of DAPT.

### Limitations

Our study has some limitations that need to be mentioned. First, the number of patients is limited and, although accounted for with multivariate analysis, differences in the use of anticoagulants between both groups (i.e., the introduction of bivalirudin) and the shift from femoral to radial approach for PCI might have influenced the results. The risk of selection bias with respect to clopidogrel or ticagrelor treatment, however, was minimal because in all ambulances in our region only one of the antiplatelet drugs was available at a time. Second, due to the study design as a retrospective registry, events might have been missed if they were not recorded in the patient file, although it is unlikely that thrombotic events and the more severe bleeding events would have been missed. Third, our registry contains all patients receiving an antiplatelet loading dose instead of selecting patients with STEMI only, thereby resembling everyday practice. The interaction subgroup analysis did not suggest our findings to be different for STEMI patients or patients without a cardiac-related diagnosis. Last, it would have been interesting to compare our data to a group of patients who received prasugrel or no antiplatelet treatment in the ambulance, but such patient groups were not available in our region.

## Conclusion

In this registry, using real-world data, no significant differences were found for thrombotic or bleeding events after administration of a ticagrelor versus a clopidogrel loading dose in patients treated according to STEMI protocol in the prehospital setting. Although it is still unclear if prehospital administration of a P2Y _12_ inhibitor is beneficial, there seems to be no safety risk associated with prehospital initiation of ticagrelor compared with clopidogrel.
